# Characterization and investigation of biological properties of silver nanoparticle-doped hydroxyapatite-based surfaces on zirconium

**DOI:** 10.1038/s41598-023-33992-5

**Published:** 2023-04-25

**Authors:** Tuba Yildiz, Salih Durdu, Kadriye Ozcan, Metin Usta

**Affiliations:** 1 Materials Science and Engineering, Gebze Technical University, 41400 Gebze, Turkey; 2 Industrial Engineering, Giresun University, 28200 Giresun, Turkey; 3 Genetics and Bioengineering, Giresun University, 28200 Giresun, Turkey; 4 Aluminum Research Center (GTU-AAUM), Gebze Technical University, 41400 Gebze, Turkey; 5 Faculty of Engineering, Giresun University, 28200 Giresun, Turkey

**Keywords:** Biomaterials, Biomaterials

## Abstract

The infections leading to failed implants can be controlled mainly by metal and metal oxide-based nanoparticles. In this work, the randomly distributed AgNPs-doped onto hydroxyapatite-based surfaces were produced on zirconium by micro arc oxidation (MAO) and electrochemical deposition processes. The surfaces were characterized by XRD, SEM, EDX mapping and EDX area and contact angle goniometer. AgNPs-doped MAO surfaces, which is beneficial for bone tissue growth exhibited hydrophilic behaviors. The bioactivity of the AgNPs-doped MAO surfaces is improved compared to bare Zr substrate under SBF conditions. Importantly, the AgNPs-doped MAO surfaces exhibited antimicrobial activity for *E. coli* and *S. aureus* compared to control samples.

## Introduction

Zirconium (Zr) and its alloys possess superior chemical and mechanical properties such as chemical stability, good corrosion resistance, and low toxicity^1,2^. Therefore, they are usually preferred in the nuclear industry, chemical engineering, and orthopedic and dental implants^3–5^. Due to the presence of a natural passive oxide layer on the surface, Zr releases fewer ions than Ti in physiological solutions such as Hank’ and SBF. The natural oxide layer (ZrO_2_) protects against corrosion^6,7^. The modulus of elasticity of Ti alloys (100–110 GPa), CoCr alloys (230 GPa) and stainless steel (189–205 GPa) in interacting with bone tissue is very high compared to bone structure (10–30 GPa). This resulted in weakening of the bone during treatment^8^. Since Young’s modulus of Zr (92 GPa) is lower than that of CoCr, stainless steel, and Ti and their alloys, it causes a minimum stress-shielding effect with bone. Therefore, Zr is one of the potential implant materials of the future due to its superior properties^9,10^. However, Zr is not chemically or biologically bound to bone tissue due to its bio inertness. Since the formation of bone tissue between the bone and Zr interface cannot be observed chemically or biologically, this region is usually encapsulated by fibrous tissue after implantation^11^. The bioactivity at the surface of Zr and its alloys triggers new bone formation by improving biocompatibility and corrosion resistance^12–14^. To overcome these disadvantages, ZrO_2_- and/or hydroxyapatite-based coatings are fabricated on Zr and its alloys^2,6,8,15–17^. Thus, bioactivity and osseointegration ability are increased and recovery time is shortened.

Hydroxyapatite (HA) is a biocompatible and bioactive bioceramic that can chemically bond and interact with surrounding tissues^18^. This inorganic phosphate is applied to metallic biomaterials due to its chemical and structural similarity to human bone. HA is described as osteoconductive due to its strong bonding ability with natural bone tissue^19,20^.

Microorganisms that naturally live in or on human skin can easily infect an implant or insertion site during surgery^21^. Common postoperative infections observed after implantation are due to microorganisms in implanted prostheses^22^. Gram-positive and Gram-negative bacterial strains are the main causes of orthopedic infections^23^. Systemic antibiotics administered in clinical practice have failed to support the treatment of implant-associated infections. Once an infection occurs, it is usually cleared by removal of the implant. An effective method of preventing infection is to prevent the initial adherence of bacteria to the implant surface^24^. Therefore, implant surfaces should be coated with antibacterial structures such as Ag, Cu and Zn^25^.

Silver (Ag) is an important antibacterial agent that prevents microbial colonization. Furthermore, it exhibits biocompatibility and non-toxicity to human cells at low concentrations^26–28^. Ag may continue to show antibacterial properties after antibiotics are depleted and may increase the antifungal activity of antibiotics^29^. There are also studies using systemic antibiotic therapy and local delivery of Ag nanoparticles (AgNP) in vitro and in vivo. The results show that AgNPs increase the antibacterial efficacy compared to antibiotics and reduce the usage of antibiotics^30,31^.

The exact mechanisms of nanoparticle (NPs) toxicity to various bacteria are unclear. NPs can adhere to the bacterial membrane by electrostatic interaction and disrupt the integrity of the bacterial membrane. Nanotoxicity is usually triggered by the induction of oxidative stress with the formation of free radicals, i.e., reactive oxygen species, after nanoparticle application. Most importantly, compared to antibiotics, nanoparticles can effectively prevent microbial drug resistance in certain situations. Widespread use of antibiotics has created numerous public health hazards, such as super-drugs that do not respond to any available drug, and epidemics where the drug is not advocated. The search for new and effective bactericidal materials is important in combating drug resistance. Thus, NPs have been identified as a promising approach to overcome this problem. NPs are an effective therapeutic method in the fight against microbial resistance and multidrug-resistant mutants. In addition, it has gained importance as an anti-bacterial agent in recent years because it overcomes antibiotic resistance mechanisms and fights microbes using multiple mechanisms^32–34^.

In recent years, there have been some studies on ZrO_2_ and/or HA coatings coated with MAO on zirconium and its alloys^8,10,12,17,35^. To improve the antibacterial ability of bioactive and biocompatible MAO coatings on zirconium, there are very limited researches on the preparation of MAO coatings with Ag ions/Ag layer/AgNPs^36–38^. Fidan et al.^37^ fabricated Ag ions-doped ZrO_2_ layer on zirconium by one-step MAO and investigated the antimicrobial activity against MRSA suspension. Durdu et al.^36^ produced antibacterial Ag-nanolayers a thickness of 20 nm from bioceramic coatings formed by combined MAO and PVD techniques on zirconium and investigated the in vitro properties. Oleshko et al. synthesized AgNPs-decorated ZrO_2_ coatings on ZrNb alloys by adding AgNPs to the electrolyte in a one-step MAO and investigated the antimicrobial activity for *S. aureus*^38^. However, antibacterial NPs structure must be formed on the outer layer because bacteria first contact the outer layer of the surfaces. Therefore, the effect of AgNPs on the MAO coatings should be studied in detail.

The MAO process performed in an alkaline electrolyte is an electrochemical process. Ceramic-like coatings with porous, homogeneous, hard, wear-resistant, corrosion-resistant, heat-resistant, electrically insulating and decorative ostentatious multifunctionality are produced by the MAO process^39^. The ED technique is based on the collection and deposition of ionic substances in solutions on metallic or non-metallic substrates through electrostatic interactions. This method offers a great advantage in producing desired shapes and covering large areas^40^.

There has been no study on the production of AgNPs-doped hydroxyapatite-based coatings on Zr by combined MAO and ED processes so far. Thus, this work aims to produce antibacterial AgNPs-doped hydroxyapatite-based bioceramic surfaces on zirconium by using MAO and ED techniques for implant applications. The phase structure, morphology, elemental amount, binding energy and wettability of all surfaces were analyzed by XRD, SEM, EDX-mapping, XPS and contact angle goniometer, respectively. In vitro bioactivity was investigated by immersion test in SBF for 28 days. Furthermore, bacterial tests were carried out for *S. aureus* and *E. coli* bacteria.

## Experimental details

### Sample preparation

Pure zirconium (Zr) plates were polished up to 2000 # sandpapers. Afterwards, all prepared plates were cleaned in an ultrasonic bath and were dried.

### MAO process

**Figure 1:**
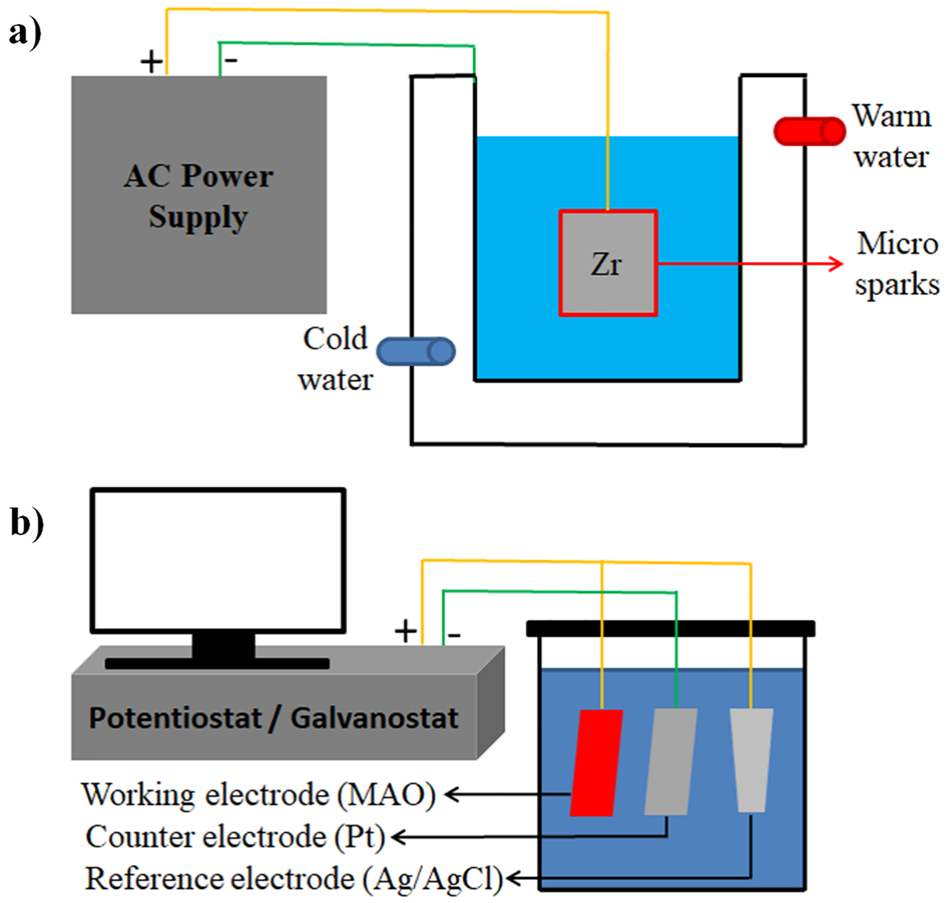
Schematic representation of experimental set up: (**a**) MAO system and (**b**) ED system.

The MAO equipment with an AC power supply contains a stainless steel container, cooling and stirring systems as shown in Fig. 1a. The Zr substrate and stainless steel container served as the anode and the cathode, respectively. The prepared electrolyte contained (CH_3_COO)_2_Ca (calcium acetate, Alfa Aesar), β-C_3_H_5_(OH_2_)PO_4_Ca (β-calcium glycerophosphate, Alfa Aesar) and deionized water. The MAO was carried out at 0.379 A/cm^2^ for 15, 30 and 45 min below 30 °C. After the MAO, all samples were cleaned in an ultrasonic bath and dried.

### ED process

Antibacterial AgNPs on the MAO surfaces were deposited at a constant potential value of -1 V for 0.5 min by using a potentiostat/galvanostat device (Metrohm Autolab). All MAO surfaces were coated in AgNO_3_ (Merck) based aqueous electrolyte. The AgNPs structure was randomly accumulated on the MAO surfaces by the ED process. After the ED process, all samples were cleaned in an ultrasonic bath and dried. Schematic representation of experimental set up of the ED system is given Fig. 1b.

### Characterization of the surfaces

Phase structure on all surfaces was detected by XRD (Bruker D8 Advance) using Cu-Kα between 20° and 80° with a step size of 0.02°/min. The average thickness of the MAO coatings was measured at 40 different points by an eddy current device (Fischer Dualscope MP40). Average roughness of the MAO coatings was evaluated by surface profilometer (Dektak 8). The morphology, elemental distribution and elemental amount of the surfaces were investigated by SEM (Philips XL20S FEG) and EDX-mapping and –area, respectively. The binding energies and surface chemistry of the surfaces were evaluated with Al–Kα radiation (1486.61 eV) by XPS (SPECS GmbH PHOIBOS 150). The average contact angles of the surfaces were investigated with a sessile constant drop technique by a Dataphysics OCA-15EC contact angle goniometer. All contact angles were measured within 1 min after water droplets with 1 µL were contacted the surfaces.

### Bioactivity test

In vitro bioactivity test was carried out by a soaking test in SBF at 36.5 °C for 10 days^41^. SBF solution was refreshed for everyday. Body temperature (36.5 °C) was maintained during immersion test in the incubator device After the SBF test was completed, all samples were washed with distilled water and dried at room temperature. All immersed surfaces were analyzed by SEM, EDX-mapping and EDX-area.

### Microbial adhesion test

Microbial adhesion experiments were carried out with *Staphylococcus aureus* and *Escherichia coli*. First, all samples were sterilized in an autoclave. Then, AgNPs-coated MAO and bare Zr samples were treated with test microorganisms adjusted according to 0.5 McFarland scale. For this process, the samples of sizes with 10 mm × 10 mm × 1 mm were immersed in 5 mL of MHB medium. After incubation at 37 °C at 125 rpm for 24 h on an orbital shaker, the samples were removed from the medium and washed with 15 mL of water to remove non-adherent organisms. This process was repeated for 3 times. Then, each sample was taken into a clean tube and 2 mL of 150 mM NaCl was added and vortexed for 2 min to collect the bacteria attached to its surface. Serial dilutions of the obtained bacterial solution were made and 100 µL were taken from the dilutions and applied to MHA medium by spreading method. At the end of 48 h of incubation at 37 °C, colony count was made and % inhibition was calculated. All experiments were performed in triplicate.

### Statistical analysis

Statistical analysis was carried out by “IBM SPSS Statistics 22 SP” software. All data were reported as standard deviation (mean ± SD). Statistical significance between the means was decided by one-way ANOVA and Duncan’s test, *p* < 0.05 was considered statistically important.

## Results and discussion

**Figure 2:**
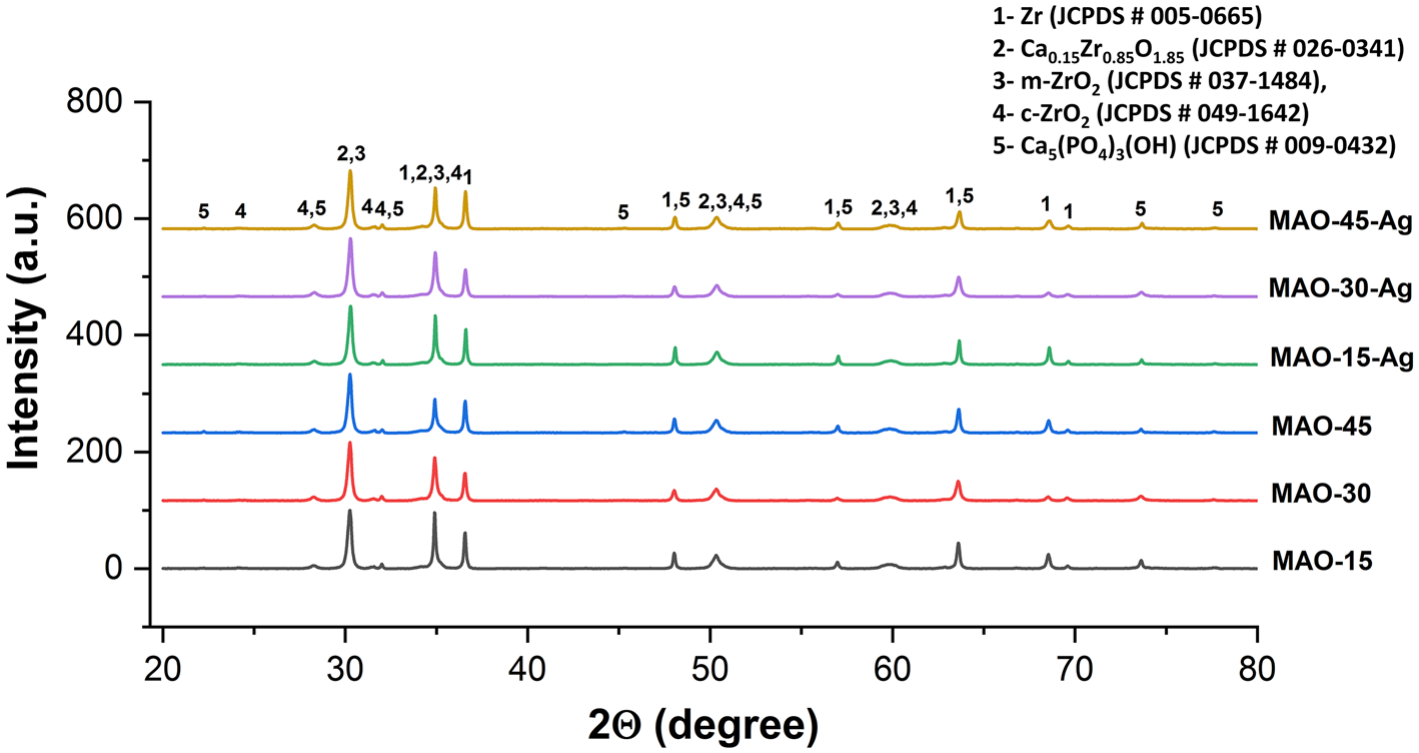
XRD spectra of the MAO and AgNPs-doped MAO surfaces.

The phase structures of all MAO and AgNPs-doped MAO coatings are illustrated in Fig. 2. As shown in XRD spectra, bioceramic composite phases such as Zr (zirconium, JCPDS # 005–0665), m-ZrO_2_ (monoclinic ZrO_2_, JCPDS # 037–1484), c-ZrO_2_ (cubic ZrO_2_, JCPDS # 049–1642), Ca_0.15_Zr_0.85_O_1.85_ (calcium zirconium oxide, JCPDS # 026–0341) and Ca_5_(PO_4_)_3_(OH) (hydroxyapatite, JCPDS # 009–0432) are observed on the MAO and AgNPs-doped MAO coatings. The Zr detected on the surfaces may be originated in substrate and/or unreacted metallic structure in the coating structures. The m-ZrO_2_ as minor phase and c-ZrO_2_ as major phase are observed in all coatings. Cationic Zr^4+^, anionic OH^−^ and H_2_O react in discharge channels due to the electrostatic interactions. Subsequently, ZrO_2_ is syntheses structure through the MAO process^42^. The c-ZrO_2_ is stable phase observed as major on the MAO surfaces since instantaneous temperature reached up to 10^6^ K in discharge channels during the MAO^9^. With increasing MAO treatment time, the intensity of hydroxyapatite increases while the intensity of the metallic Zr relatively decreases. The formation mechanism of hydroxyapatite was discussed in detail in previous works^8,17,35,43–45^. The partially stabilized Ca_0.15_Zr_0.85_O_1.85_ was formed on the MAO surfaces by the corporation Ca^2+^ and c-ZrO_2_. HAP structure easily occurs on zirconium oxide due to the catalytic effect of free radical Zr–OH groups on surface^16^. Cationic Ca^2+^ and anionic PO_4_^3−^ ionized from calcium acetate and β-calcium glycerophosphate-based electrolyte react with H_2_O molecules. Subsequently, they form bioactive and biocompatible hydroxyapatite during MAO process^46–48^. However, the elemental or compound phase structure of Ag could not be detected by XRD due to the existence of a trace amount of AgNPs on the MAO surfaces. Thus, XPS and EDX analyses were carried out to prove the existence of elemental and phase structure of AgNPs on the MAO surfaces.

**Figure 3:**
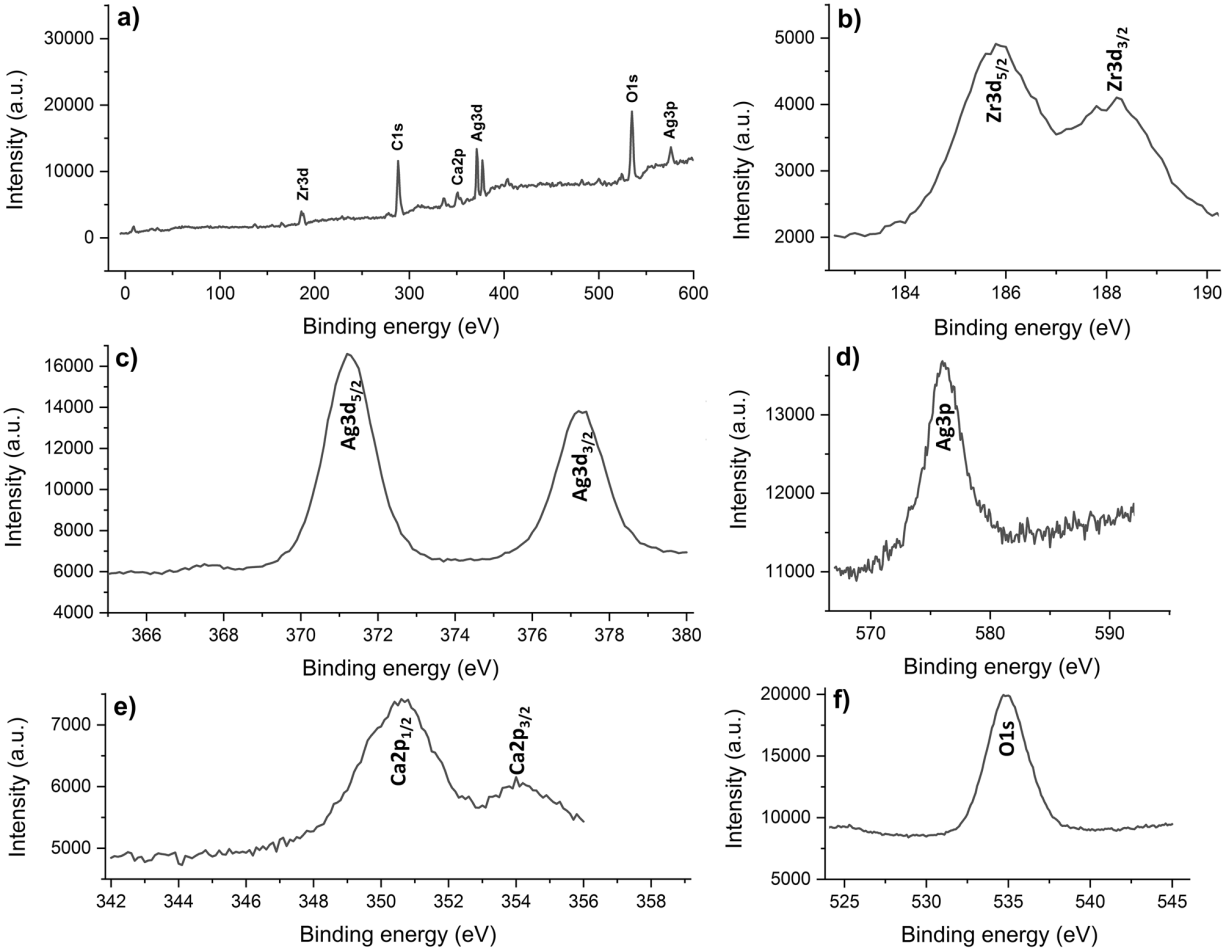
XPS spectra of AgNPs-doped MAO surface produced at 15 min: (**a**) Survey, (**b**) Zr3d, (**c**) Ag3d, (**d**) Ag3p, (**e**) Ca2p and (**f**) O1s.

The XPS analysis was performed to determine the binding energies and surface chemistry of the elements in the structure of AgNPs-doped MAO coating produced at 15 min. XPS survey and XPS spectra of Ag3p, Ag3d, Ca2p, O1s and Zr3d on AgNPs-doped MAO surface are shown in Fig. 3. According to the XPS survey spectrum, the AgNPs-doped MAO coatings contain Ca, P, O, Zr and Ag. These elements are also detected by EDX-mapping and EDX-area. The C1s spectrum is observed owing to the surface contamination during handling and cleaning. The Zr3d spectrum consists of two peaks at the binding energy of 185.9 eV for Zr3d_5/2_ and at the binding energy of 188.3 eV for Zr3d_3/2_. Double Zr3d peaks in XPS spectra correspond to the presence of ZrO_2_^49,50^. The O1s spectrum reveals a single peak at the binding energy of 534.9 eV. The Ca2p spectrum contains double peaks at the binding energies of 350.7 and 353.9 eV. Double Ca2p peaks in XPS spectra refer to the existence of hydroxyapatite^35,51^. The Ag3d spectrum contains double peaks at the binding energies of 371.1 eV and 377.2 eV. Moreover, The Ag3p spectrum reveals a single peak at the binding energy of 575.8 eV. This indicates the existence of AgNPs on the MAO surface as supported in the literature, SEM and EDX results^52,53^.

**Figure 4:**
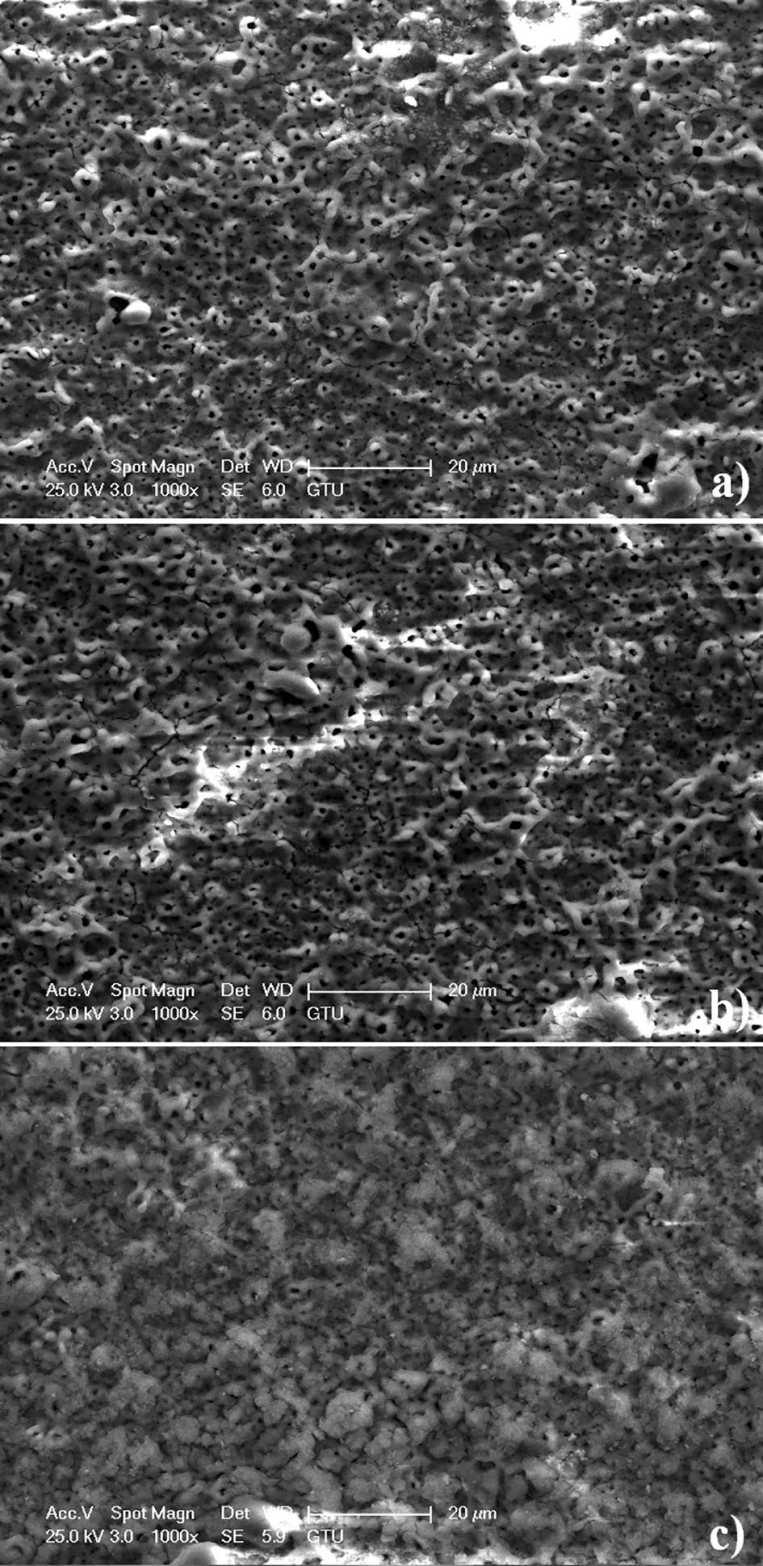
Surface topography of the MAO surfaces taken at low magnifications: (**a**) 15 min, (**b**) 30 min and (**c**) 45 min.

To distinguish surface structure, the surface morphology of the MAO coatings taken at low magnifications is given in Fig. 4. Typically, porous and rough MAO morphology occur on Zr substrates, which is due to the presence of micro sparks by MAO process. Following the breakdown of the dielectric film, micro sparks begin to form where the oxide film coating is weak. There are many large and small pores on the coating surface. This causes an increase in the micro-discharge channels formed during the process. With increasing time, larger sized discharge channels are formed. The reason for the expansion of the discharge channels is to reduce the pressure inside the discharge channels with the effect of increasing time during the MAO process. Large discharge channels eliminate preformed small discharge channels on the surface. As a result, with increasing time, there is a high anodic potential transition under galvanostatic conditions throughout the MAO process. Thus, while the discharge channels decrease in number, their size increases. As a result, a rough and porous surface is produced. The micro pores on the surfaces are called as discharge channels (micro discharge channels). Usually, the size of discharge channels improves while the number of it decreases with increasing MAO treatment time. The average roughnesses of the MAO surfaces for 15, 30 and 45 min are obtained as 0.65, 0.61 and 0.89 µm, respectively. However, the MAO process reaches saturation point above 30 min. The micropores on the MAO coating produced at 45 min are enclosed compared to low treatment time, as seen in Fig. 4c. The average thickness values for 15, 30 and 45 min are measured as 57.6, 58.3 and 58.7 µm, respectively. The kinetic rate of the MAO process slows down above 30 min. Thus, the formation of the MAO begins to stop. In the direction of these, the MAO surface may completely deteriorate above 45 min.

**Figure 5:**
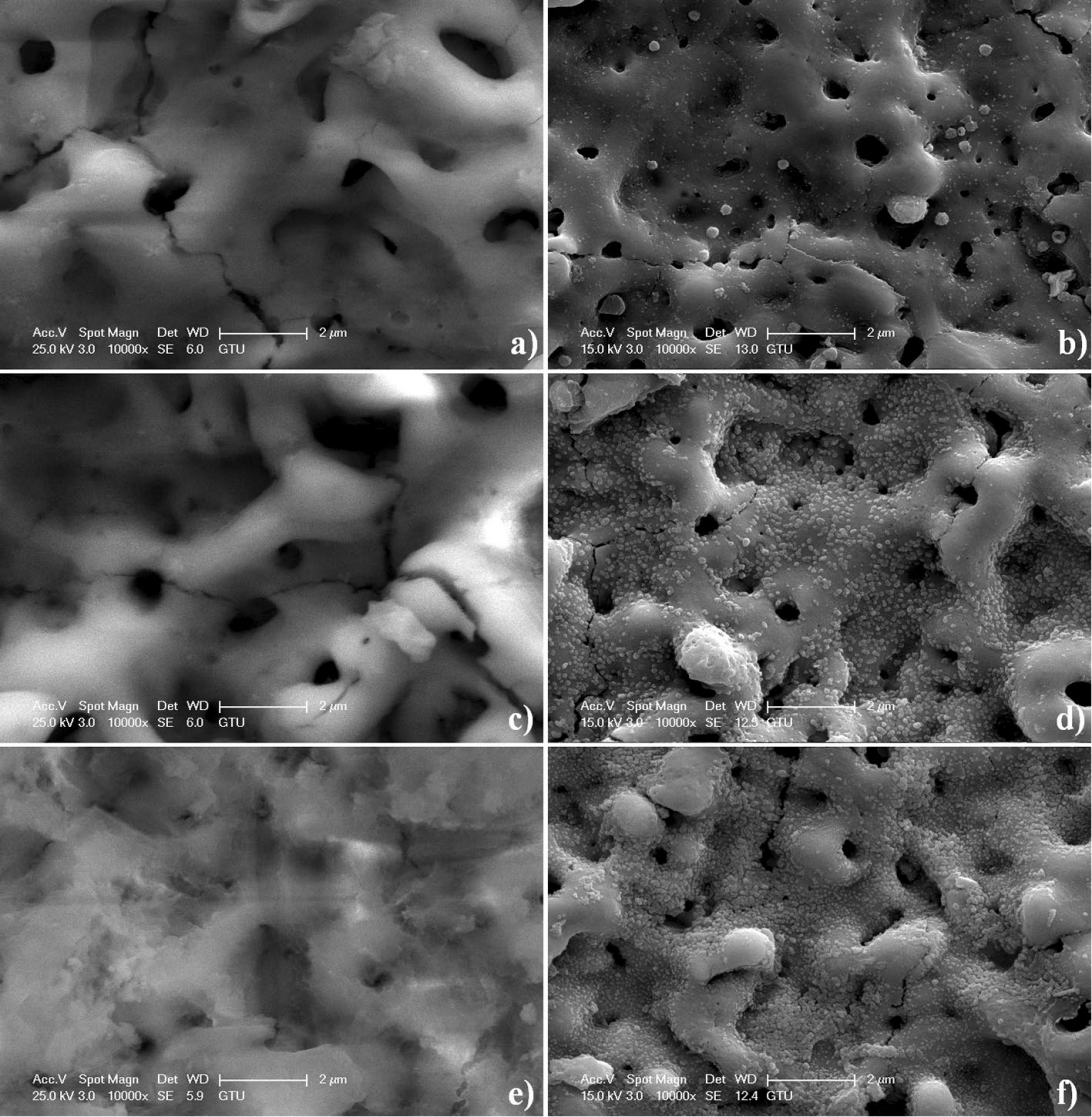
Surface topography of the MAO and AgNPs-doped MAO surfaces taken at high magnifications: (**a**) MAO-15, (**b**) MAO-15-Ag, (**c**) MAO-30, (**d**) MAO-30-Ag, (**e**) MAO-45 and (**d**) MAO-45-Ag.

The surface morphology of AgNPs-doped MAO surfaces is seen in Fig. 5. The AgNPs structures are randomly and uniformly accumulated on the MAO surfaces. Furthermore, the AgNPs structures do not significantly change the morphology of MAO. The porous morphology on MAO is maintained by the ED process. The ED process is applied to all MAO surfaces by using identical experimental conditions such as voltage, electrolyte and time. However, the size and amount of AgNPs on the MAO surfaces increase from 15 to 45 min. The number of nucleation and growth sites for Ag structure increases on the MAO surfaces since the porosity and roughness of the surfaces enhance with increasing MAO treatment time. Also, anionic OH^-^ ions on free radical Zr–OH groups may contribute to migrate cationic Ag^+^ ions onto the surface. Compared to low treatment time, more Ag^+^ ions can diffuse on negatively charged OH^-^ on the MAO surface since the intensity of c-ZrO_2_ increases with increasing MAO treatment time, as seen in Fig. 2. Eventually, more AgNPs are accumulated onto the MAO surfaces with increasing MAO treatment time when the ED treatment time keeps constant.

**Figure 6:**
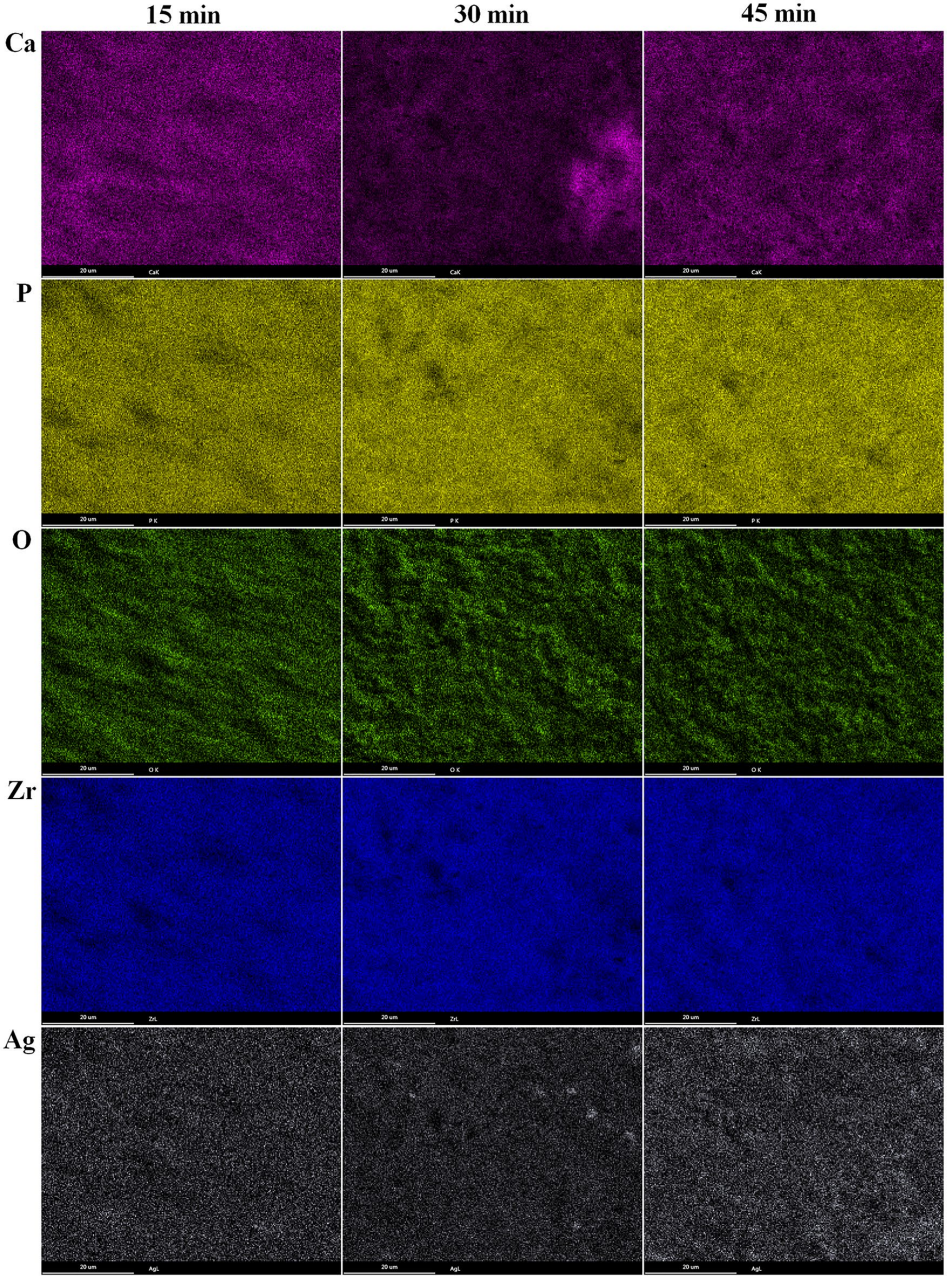
EDX-mapping images of AgNPs-doped MAO surfaces.

The elemental distribution of AgNPs-doped MAO surfaces is shown in Fig. 6. As expected, the Ca, P, O, Zr and Ag elements are observed on the surfaces. Moreover, all elements are homogenously distributed throughout the whole surface. The dark sites on the surfaces refer to discharge channels. The Ca, P, O and Zr elements are directly related with the phases m-ZrO_2_, c-ZrO_2_, Ca_0.15_Zr_0.85_O_1.85_ and Ca_5_(PO_4_)_3_(OH) phases coming from the MAO surface. Furthermore, the elemental Ag is uniformly distributed through the surfaces whereas the elemental or compound phase structure of Ag is not detected by XRD. The elemental amount of the surfaces is given in Table 1.

**Table 1:** EDX area results of the MAO and AgNPs-doped MAO surfaces.

Element	15 min	30 min	45 min	15 min—Ag	30 min—Ag	45 min—Ag
	At.%	At.%	At.%	At.%	At.%	At.%
Ca	4.98	5.45	8.31	5.69	6.49	6.00
P	6.30	6.03	6.59	5.96	4.84	4.82
O	71.10	72.02	74.00	61.42	58.62	58.69
Zr	17.62	16.45	11.09	26.73	29.42	29.89
Ag	–	–	–	0.19	0.63	0.60

**Figure 7:**
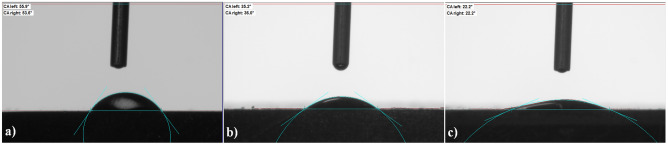
Contact angle images of the surfaces: (**a**) bare Zr, (**b**) MAO-30 and (**c**) MAO-30-Ag.

The wettability of bare Zr, bare MAO surfaces and AgNPs-doped MAO surfaces was investigated by contact angle goniometer as seen in Table 2 and Fig. 7. The contact angle of 65° is used to express the difference between hydrophilic and hydrophobic surface^54^. The bare Zr surface indicates a hydrophilic character since the average contact angle is lower than 65°. However, the average contact angles of the MAO surface are lower than one of bare Zr. The hydrophilic ability of the MAO surface is improved with increasing MAO treatment time. The contact angles of droplets on solid surfaces depend on some parameters such as the surface chemistry, topography and roughness^55,56^. Compared to bare Zr substrates, the water molecules on the MAO surfaces are easily adsorbed since the surface of MAO coatings is porous. Furthermore, one of the most important critical factors is the existence of OH^-^ group on the surface of coating. It is well known that a high amount of OH^–^ is associated with improving hydrophilicity^57^. The intensity of c-ZrO_2_ improves with increasing treatment time, as seen in Fig. 2. This refers to improve polarity of surface due to the existence of Zr–OH groups on the MAO surfaces. Polar surfaces improve wettability and exhibit low contact angles^58^. However, AgNPs-doped MAO surfaces indicate very low contact angles respect to the MAO surfaces. It is well known that a hydrophilic surface tends to enhance cell adhesion, cell differentiation, bone mineralization and biological activity^59^.

**Table 2:** Average contact angles of the surfaces.

Bare Zr	15 min	30 min	45 min	15 min—Ag	30 min—Ag	45 min—Ag
54.8 ± 1.2	54.2 ± 0.7	35.6 ± 0.4	25.2 ± 0.1	13.7 ± 0.5	22.2 ± 0.0	16.1 ± 0.0

**Figure 8:**
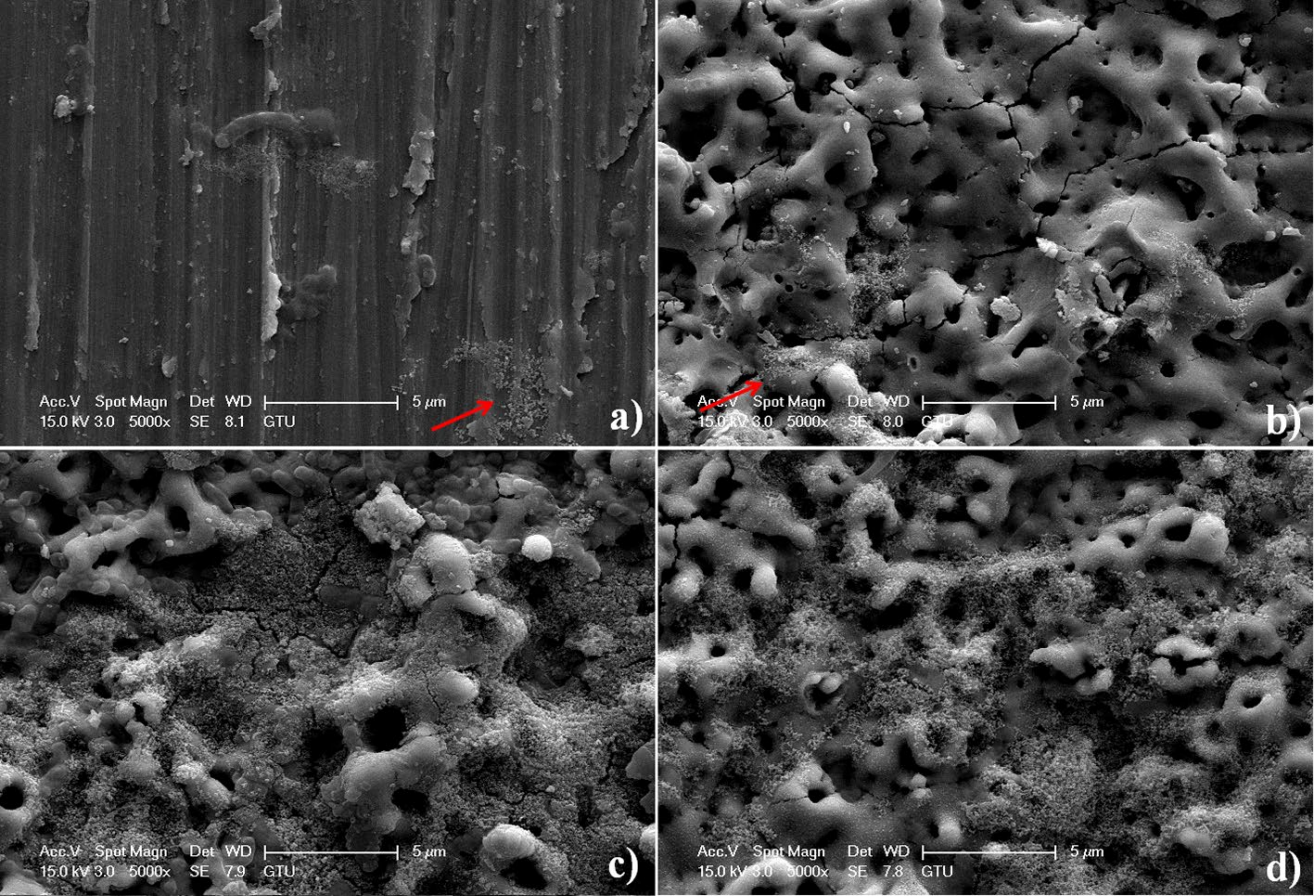
Surface topography of the surfaces at post-immersion in SBF for 10 days: (**a**) bare Zr, (**b**) MAO-15-Ag, (**c**) MAO-30-Ag and (**d**) MAO-45-Ag.

**Figure 9:**
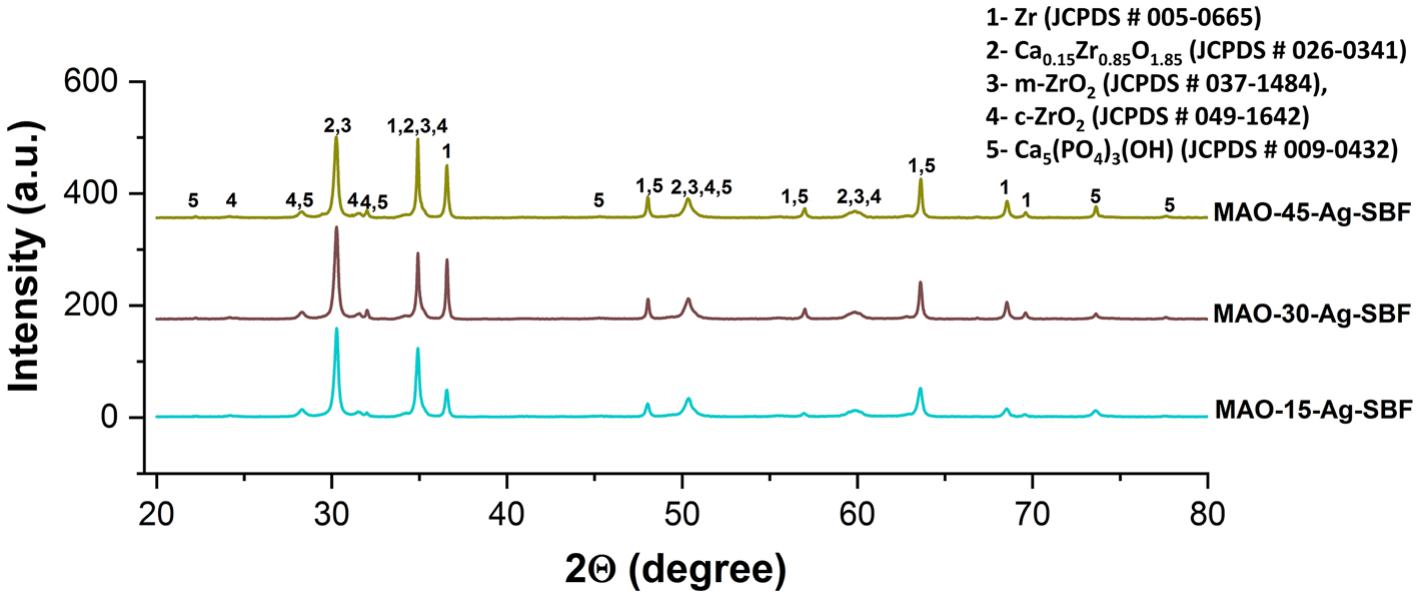
XRD spectra of AgNPs-doped MAO surfaces at post immersion in SBF.

In vitro immersion test in SBF is typically carried out to estimate the predicting bioactivity of a biomaterial surface. To analyze the bioactivity of bare Zr and AgNPs-doped MAO surfaces, in vitro immersion test was carried out at 36.5 °C under SBF for 10 days. The surface morphology and phase composition of bare Zr and AgNPs-doped MAO surfaces after immersion test are shown in Figs. 8, 9, respectively. Red arrows on Fig. 8 indicate newly formed secondary particles on bare and AgNPs-doped MAO surfaces at post-immersion in SBF. As shown in Fig. 9, bioceramic composite phases such as Zr (zirconium, JCPDS # 005-0665), m-ZrO_2_ (monoclinic ZrO_2_, JCPDS # 037-1484), c-ZrO_2_ (cubic ZrO_2_, JCPDS # 049-1642), Ca_0.15_Zr_0.85_O_1.85_ (calcium zirconium oxide, JCPDS # 026-0341) and Ca_5_(PO_4_)_3_(OH) (hydroxyapatite, JCPDS # 009-0432) are observed on the MAO and AgNPs-doped MAO coatings at post-immersion in SBF. The spherically shaped secondary particles, which are randomly distributed, are observed on the surfaces. These secondary particles refer to the existence of a hydroxyapatite (secondary apatite) structure on the surface. The low amount of secondary apatite structure is locally accumulated on bare Zr surface due to bioinert nature of it. However, more homogenously secondary apatite deposition on AgNPs-doped MAO surfaces reveals with respect to bare Zr substrate owing to the presence of hydroxyapatite, ZrO_2_ and Ag contents. The hydroxyapatite, ZrO_2_ and Ag contents on AgNPs-doped MAO surfaces trigger the diffusion of cationic Ca^2+^ and anionic PO_4_^3−^ ions in SBF with the electrostatic interactions^60^. The ZrO_2_ provide effective epitaxial nucleation sites for secondary apatite structures under SBF^61^. Therefore, it is believed that the enhanced secondary apatite-forming ability of the ZrO_2_ is related with the abundant Zr–OH groups on the MAO surfaces. Especially, Zr–OH groups trigger the absorption of anionic PO_4_^3−^ by the cationic Ca^2+^ in SBF, which contributes to the electrostatic potential interaction between secondary apatite nuclei and outer surface. Moreover, it is assumed that some of Ca^2+^ sites in the apatite lattice were substituted with Ag^+^ ions during SBF. Thus, this accelerates the dissolution rate of secondary apatite in SBF solution^62^. The faster dissolution of soluble ions to the SBF escalates the apatite formation and precipitation on AgNPs-doped MAO surfaces. Eventually, this results on the formation of more secondary apatite on AgNPs-doped MAO surfaces with respect to bare Zr surface.

**Figure 10:**
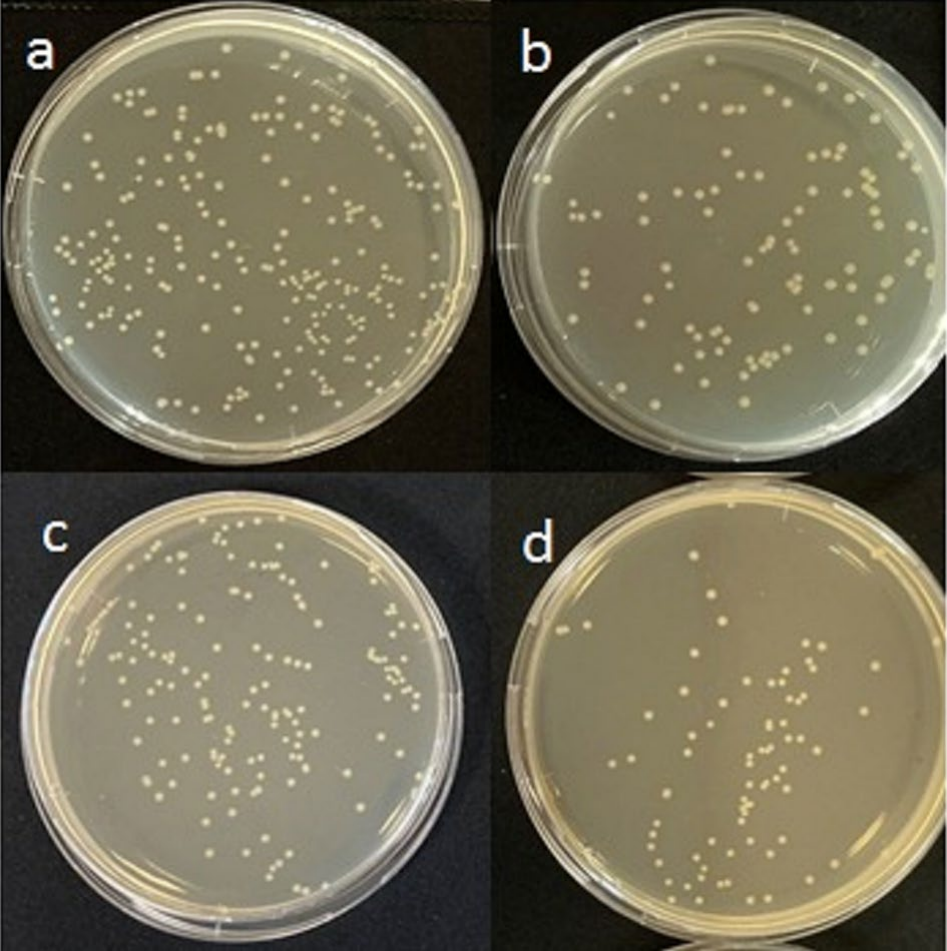
Reduction in microbial colonies after re-culturation in samples with the highest antibacterial activity on MAO-45-Ag: (**a**) *E. coli* viability after re-culture on bare Zr surfaces, (**b**) *E. coli* viability after re-culture on MAO-45-Ag surfaces, (**c**) *S. aureus* viability after re-culture on bare Zr surfaces, (**b**) *S. aureus* viability after re-culture on MAO-45-Ag surfaces.

For antimicrobial analysis of bare Zr and AgNPs-doped MAO surfaces, *S. aureus* and *E. coli* bacteria adhering to the surface treated with microorganisms were collected and re-cultured. After incubation, the reduction in bacterial viability is calculated as percent inhibition, as given in Fig. 10 and Table 3. *S. aureus* that is the most common organism associated with infections is one of nosocomial infections. *S. aureus* is a combination of microbial immuno-evasive strategies. Thus, *S. aureus* is often the cause of wound infections at post-surgical operations^63^. Also, *E. coli* is one of the main reasons of Gram-negative orthopedic implant infections. *E. coli* is involved in various extra-intestinal diseases such as urinary tract infections and bacteremia that could result in orthopedic implant seeding^64,65^. AgNP-doped MAO surfaces show antimicrobial activity in the range of 15.4–59.2% compared to bare Zr substrate for *E. coli* and *S. aureus*. The best inhibition is detected against *S. aureus* on the MAO-45-Ag. The antimicrobial activity on the AgNPs-doped MAO surfaces increased for *E. coli* and *S. aureus* organisms. In general, for identical surfaces, antimicrobial activity to *S. aureus* is greater than to *E. coli*. The order of antimicrobial efficiency is observed as MAO-45-Ag > MAO-30-Ag > MAO-15-Ag. This result is also supported in Fig. 5. The amount of Ag on the MAO produced at 30 min can be slightly higher than 45 min since the AgNPs structures are not detected by EDX in dark regions (discharge channels). However, according to the SEM images, the distribution of AgNPs on the MAO surface produced at 45 min is more than ones at 15 min and 30 min. Thus, antimicrobial efficiency of the AgNPs-doped MAO produced at 45 min is the best obtained within all samples.

**Table 3:** Bacterial adhesion to the samples and percentage of adhesion inhibition.

Samples	*E. coli*		*S. aureus*	
	CFU 10^4^/mL	Inhibition%	CFU 10^4^/mL	Inhibition%
MAO-15-Ag	1.37 ± 0.15^c^	15.4	1.30 ± 0.12^de^	23.1
MAO-30-Ag	1.34 ± 0.10^ cd^	17.3	1.25 ± 0.09f.	26.0
MAO-45-Ag	0.98 ± 0.06^ g^	39.5	0.74 ± 0.07^ h^	56.2
Zr (Control)	1.62 ± 0.10^b^	–	1.69 ± 0.15^a^	–

^a–h^Statistical significance between means was assessed using one-way analysis of variance followed by Duncan's test as a post-analysis of variance test. Data are presented as mean and standard deviation (SD). The data shown with the same letters are statistically insignificant (*p* > 0.05), while those indicated with different letters are statistically significant (*p* < 0.05).

Ag indicating broad spectrum antimicrobial effect at very low concentrations possesses many advantages such as biocompatibility and good antibacterial ability^66^. Ag passes through the microbial cell wall and can bind to DNA. It can interfere with the replication process. In the literature, the focus in this area is the development of Ag-substituted HA coatings to minimize the adhesion of bacteria^67^. Thus, Ag structure that exhibits a broad spectrum of antimicrobial activity is often preferred for antibacterial purposes in the medical field such as burn creams, vascular grafts and implants^27,37,68^. Oleshko et al.^38^ obtained excellent antimicrobial effect the AgNPs-decorated MAO surfaces on ZrNb alloy for *S. aureus*. For *E. coli*, antimicrobial effect on the Ag-nanolayer on the MAO surfaces on Zr is observed compared to control samples^36^.

## Conclusion

In this work, the randomly distributed AgNPs-doped hydroxyapatite-based bioceramic composite MAO surfaces were fabricated on Zr by combined MAO and ED processes. Bioactive and biocompatible hydroxyapatite-based bioceramic structures were detected on the surface. Furthermore, the existence of AgNPs on MAO was verified by XPS, SEM and EDX. The AgNPs-doped MAO surfaces, which are beneficial to bone structures at post-implantation, were porous and rough. Furthermore, the hydrophilicity of the AgNPs-doped MAO surfaces is significantly improved compared to bare Zr and MAO surfaces. The bioactivity of AgNPs-doped MAO surfaces is improved compared to bare Zr substrate under SBF conditions. Importantly, the AgNPs-doped MAO surfaces indicated antibacterial activity for *S. aureus* and *E. coli*. Eventually, it can be concluded that the AgNPs-deposited MAO surfaces have potential for long-term usage of implant applications.

## Data Availability

The datasets used and/or analyzed during the current study are available from the corresponding author on reasonable request.
